# An S-cone circuit for edge detection in the primate retina

**DOI:** 10.1038/s41598-019-48042-2

**Published:** 2019-08-15

**Authors:** Sara S. Patterson, James A. Kuchenbecker, James R. Anderson, Andrea S. Bordt, David W. Marshak, Maureen Neitz, Jay Neitz

**Affiliations:** 1 Graduate Program in Neuroscience, University of Washington, Seattle, WA 98109 USA; 2 Department of Ophthalmology, University of Washington, Seattle, WA 98109 USA; 3 Department of Ophthalmology and Visual Sciences, John A. Moran Eye Center, University of Utah School of Medicine, Salt Lake City, UT 84132 USA; 4 Department of Neurobiology and Anatomy, McGovern Medical School, Houston, TX 77030 USA

**Keywords:** Colour vision, Retina

## Abstract

Midget retinal ganglion cells (RGCs) are the most common RGC type in the primate retina. Their responses have been proposed to mediate both color and spatial vision, yet the specific links between midget RGC responses and visual perception are unclear. Previous research on the dual roles of midget RGCs has focused on those comparing long (L) vs. middle (M) wavelength sensitive cones. However, there is evidence for several other rare midget RGC subtypes receiving S-cone input, but their role in color and spatial vision is uncertain. Here, we confirm the existence of the single S-cone center OFF midget RGC circuit in the central retina of macaque monkey both structurally and functionally. We investigated the receptive field properties of the S-OFF midget circuit with single cell electrophysiology and 3D electron microscopy reconstructions of the upstream circuitry. Like the well-studied L vs. M midget RGCs, the S-OFF midget RGCs have a center-surround receptive field consistent with a role in spatial vision. While spectral opponency in a primate RGC is classically assumed to contribute to hue perception, a role supporting edge detection is more consistent with the S-OFF midget RGC receptive field structure and studies of hue perception.

## Introduction

Anatomical evidence for S-OFF midget RGCs is seen in the macaque (Old World) monkeys^1–4^, (but see Kolb *et al*.^5^) but not in New World monkeys^6,7^. Multi-electrode recordings in the macaque far peripheral retina find weak S-cone input to OFF midget RGCs, consistent with non-selective input from S-OFF and L/M-OFF midget bipolar cells^8^; however, pure S-cone center, OFF midget RGCs have remained elusive in single cell electrophysiology^9–11^ and electroretinography^12,13^. The discrepancies in evidence for S-OFF midget RGCs may be due to differences in species^14^, retinal eccentricity and methodology, thus, here, we sought both anatomical and physiological confirmation in the macaque retina. Because color vision^15^, spatial acuity^16^ and midget RGC response properties vary considerably with eccentricity^17^, we focused our efforts on the central retina where our research is most relevant to human visual perception.

The existence and function of S-OFF midget RGCs is relevant to a major unsolved question: how and where color and spatial information are separated in the visual pathway. This has been proposed to be accomplished in the cortex by theoretical downstream “de-multiplexing” circuits^18,19^ (but see Kingdom & Mullen^20^) or in the retina by separate populations of RGCs for color and spatial information^21–23^. In the central retina, each L- and M-cone provides the sole direct input to an ON and OFF midget circuit, forming a “private line” pathway from single cones to the parvocellular lateral geniculate nucleus (LGN)^24^. Their center-surround receptive fields perform a simple computation: comparing a single L- or M-cone center to neighboring L/M-cones in the surround. However, this seemingly simple computation introduces a complication for neural coding that vision science has yet to resolve. The center and surround receptive fields differ in both spatial location and spectral sensitivity, creating a neuron with both spatial and spectral opponency. The result is that midget RGCs carry both spatial and spectral information, but confound the two such that from an individual midget RGC’s spike output, downstream neurons cannot distinguish between chromatic and spatial stimuli (reviewed in Patterson *et al*.^25^). The goal of this work is not only to confirm the existence of S-OFF midget RGCs but understand the details of their circuity and receptive field properties so they can be fit into a larger understanding of the role of midget RGCs in color and spatial vision.

## Results

### S-cone circuits of the outer retina

**Figure 1:**
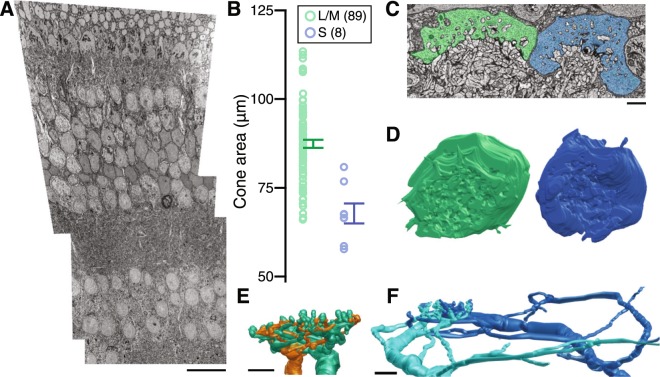
Identification of L/M- and S-cones using serial EM. (**A**) Transmission EM image of the block of tissue. Scale bar is 20 µm. (**B**) Area of L/M- and S-cone pedicles (S: 67.705 µm^2^ ± 2.81, LM: 87.359 µm^2^ ± 1.126; p = 0.0013). (**C**) Electron micrograph of neighboring LM- (green) and S-cones (blue). Scale bar is 2 µm. (**D**) 3D reconstructions of neighboring S- and L/M-cones (blue, green). (**E**) 3D reconstruction of S-ON bipolar cell dendrites at an S-cone. (**F**) 3D reconstruction of L/M-ON (teal) and L/M-OFF (orange) midget bipolar cell dendrites at an L/M-cone.

To confirm the existence of S-OFF midget RGCs using serial electron microscopy (EM; Fig. 1A), a first step was to reliably identify S-cones. We identified candidate S-cones by their small size and lack of long telendondria, branches forming gap junctions with neighboring cones^5^. While S-cones were indeed smaller than L/M-cones, distinguishing the two at any single section was difficult, especially given the changing landscape of the foveal slope in our sample (Fig. 1B–D).

Candidate S-cones were verified by reconstructing post-synaptic neurons. Cones signal changes in photon catch by modulating the rate of glutamate release from ribbon synapses onto a post-synaptic “triad” consisting of an ON-bipolar cell and two horizontal cells (reviewed by Sterling & Matthews^26^). L/M- and S-cones contact stereotyped horizontal cell and ON bipolar cell subtypes, allowing unambiguous confirmation of cone type by reconstructing the outer retina circuitry.

**Figure 2:**
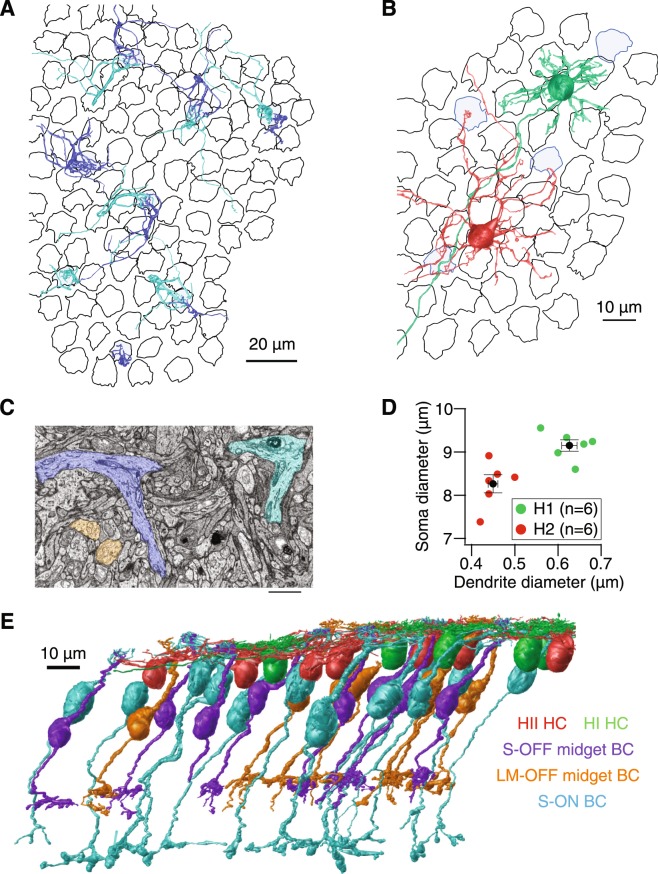
Serial EM reconstruction of the S-cone circuitry in macaque central retina. (**A**) 3D reconstructions of the dendrites of 14 S-ON bipolar cells over the cone mosaic (S-cones in blue). (**B**) 3D reconstructions demonstrate the morphological differences between HI (green) and HII (red) horizontal cells (S-cones in blue; L/M-cones in black). (**C**) Two S-ON bipolar cell dendrites (blue, cyan) converge towards an S-cone. These lateral branches distinguish S-ON bipolar cells from ON midget bipolar cells (orange) which branch directly beneath the L/M-cone pedicle. Scale bar is 2 µm. (**D**) Soma diameter plotted against primary dendrite diameter for HI and HII horizontal cells (n = 6). The dendrite diameters are 0.627 µm ± 0.018 and 0.450 µm ± 0.011 for HI and HII horizontal cells, respectively (p < 0.001). Soma diameters were 9.148 µm ± 0.134 and 8.259 µm ± 0.211 for HI and HII horizontal cells, respectively. (**E**) 3D reconstructions of the retinal neurons in this study.

In the central retina, L/M-cones are densely innervated by a single ON midget bipolar cell while S-cones provide input to several S-ON bipolar cells (Fig. 1E,F)^2,3,27^. Furthermore, S-ON bipolar cells contact multiple S-cones, forming the distinctive lateral branches shown in Fig. 2C. We reconstructed 14 S-ON bipolar cells (Fig. 2A), each contacting up to three S-cones but receiving the majority of their input from a single S-cone.

The primate retina contains two horizontal cell subtypes: HII horizontal cells preferentially contact S-cones while HI horizontal cells avoid S-cones entirely^28–30^. We reconstructed both types (Fig. 2B) and confirmed each S-cone was densely innervated by HII, but not HI horizontal cells. In past light microscopy experiments, the horizontal cell subtypes could be distinguished by their dendritic field size and cone contacts, however, applying these features to serial EM experiments requires considerable annotation efforts. To date, no complete serial EM reconstructions of primate horizontal cells have been published. We developed two criteria for early identification of horizontal cell subtypes: soma size and primary dendrite diameter (Fig. 2D). The horizontal cells identified using these criteria matched established morphological descriptions from light microscopy^31,32^.

### Each S-cone in the central retina provides the sole input to an OFF midget circuit

**Figure 3:**
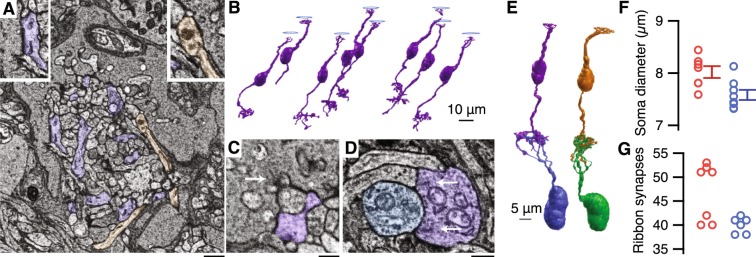
Each S-cone in the central retina provides the sole input to an OFF-midget circuit. (**A**) S-OFF midget (purple) and OFF diffuse (orange) bipolar cell processes at an S-cone. Left inset: An S-OFF midget bipolar cell basal contact. Right inset: an OFF diffuse bipolar cell basal contact. (**B**) 3D reconstructions of the S-OFF midget bipolar cells contacting eight S-cones. (**C**) An S-OFF midget bipolar cell dendrite at the triad-associated position. Scale bar is 500 nm. (**D**) An S-OFF midget bipolar cell making ribbon synapses onto an OFF midget RGC dendrite in the IPL. Scale bar is 500 nm. (**E**) Comparison of an S-cone OFF midget bipolar cell circuit (left) with an L/M-cone OFF midget circuit (right). (**F**) Soma diameters for S- and LM-cone OFF midget bipolar cells (S: 8:02 µm ± 0.11, n = 7; LM: 7.93 µm ± 0.36, n = 9, p = 0.0175). (**G**) Ribbon synapses between OFF-midget bipolar cells and OFF-midget RGCs. S-OFF midget bipolar cells made 40 ± 0.683 ribbon synapses. LM-OFF midget bipolar cells formed two groups of 39.67 ± 0.577 (n = 3) and 51.75 ± 1.291 (n = 4) ribbon synapses (expressed as Mean ± SD). The probability obtaining these two groups by chance from a single normally-distributed group was estimated using a bootstrap procedure (p = 0.0083, see Methods).

Having identified eight S-cones by morphology and postsynaptic circuitry, we next searched for OFF midget bipolar cell contacts. Indeed, a single OFF midget bipolar cell contacted each S-cone, as shown in Fig. 3B. To verify the transmission pathway from S-cones to OFF midget RGCs, S-OFF bipolar cell contacts to S-cones were characterized. We next verified that S-OFF bipolar cells provide the sole input to OFF midget RGCs and confirmed the S-cone signal is not diluted by L/M-cone input from multiple midget bipolar cells, as seen in the peripheral retina^8,33^. Six OFF midget bipolar cells contacting neighboring L/M-cones were used as controls.

Each OFF bipolar cell subtype is located at a characteristic distance from the ribbon synapse (Fig. 3C)^2,34–36^. At L/M-cones, OFF midget bipolar cell contacts are located at the “triad associated” position, adjacent to the membrane invaginations containing horizontal cell and ON bipolar cell dendrites. Virtually all S-OFF midget bipolar cell synapses were found in the same location, adjacent to the membrane invaginations containing S-ON bipolar cell dendrites (Fig. 3C). This distinguished the S-OFF midget bipolar cell dendrites from the thin, straight, OFF diffuse bipolar cell dendrites making basal contacts around the edges of the S-cone pedicle, as in Fig. 3A. The distance from the ribbon synapse shapes bipolar cell responses by defining the timing and concentration of available glutamate^37,38^. Thus, S-OFF midget bipolar cells likely have similar response properties to L/M-OFF midget bipolar cells.

Basal contacts between cone pedicles and OFF bipolar cells are recognized as membrane densities without associated pre-synaptic vesicles. The basal contacts were quantified at two S-cones, where S-OFF midget bipolar cells made 22 and 23 basal contacts. The basal contact counts are likely underestimates as the 90 nm section thickness prevented exhaustive tracing of all bipolar cell dendrites through the S-cone pedicle. However, these numbers are comparable to Klug *et al*.^1^, and to the 25 OFF midget bipolar cell basal contacts counted at a neighboring L/M-cone.

Near the fovea, each midget bipolar cell contacts a single midget RGC, forming a “private line” from a single cone to the parvocellular LGN. Outside the central retina, the number of bipolar cells converging on a single midget RGC scales with eccentricity^24,39^. We traced seven of the S-OFF midget bipolar cells to the inner retina, where each contacted a single OFF midget RGC with an average of 40 ribbon synapses (Fig. 3D, 3E, 3G). The eighth S-OFF midget bipolar cell ran off the edge of the volume. Ribbon synapses from S-OFF midget bipolar cells onto other RGCs were rarely observed.

Like the S-OFF midget bipolar cells, each L/M-OFF midget bipolar cell provided the sole input to an OFF midget RGC. Ribbon synapse counts have been reported to divide L/M-OFF midget bipolar cells into two populations, referred to as “sparsely” and “densely” branching, which presumably correspond to L- and M-cones^40–42^. The L/M-OFF midget bipolar cells in our study made an average of 39 or 51 ribbon synapses, with the sparsely branching group comparable to the S-OFF midget bipolar cells (Fig. 3G). The only anatomical difference was that S-OFF midget bipolar cells had slightly smaller somas than the LM-OFF midget bipolar cells (Fig. 3F).

Overall, we found no anatomical evidence for a significant functional difference between L/M-OFF and S-OFF midget circuits. Taken together, our reconstructions indicate the LGN indeed receives input from single S-cones through S-OFF midget RGCs in the central retina.

### S-OFF midget ganglion cells encode spatial information with center-surround receptive fields

**Figure 4:**
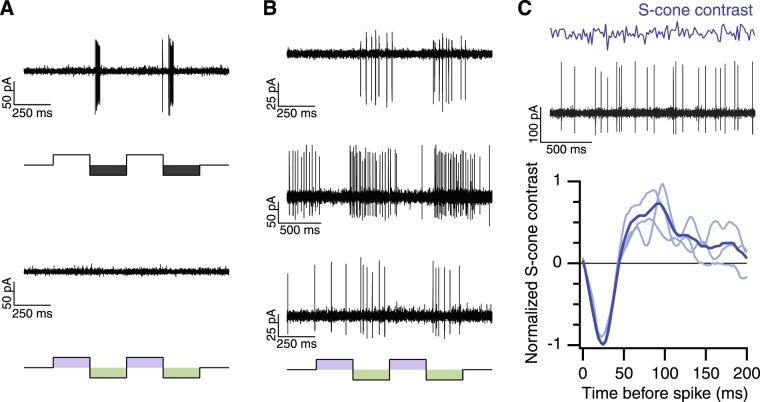
Spike responses of S-OFF midget RGCs. (**A**) Responses of three OFF midget RGCs to temporally-modulated S-cone isolating spots. (**B**) An example of the S-cone isolating Gaussian white noise (top) an S-OFF midget RGC’s response (middle). Bottom: The average linear filter (blue) from three S-OFF midget RGCs (light blue). The linear filter represents the average S-cone contrast preceding a spike.

During the course of a larger series of electrophysiology experiments on S-cone inputs to midget RGCs using our custom-built light source (see Methods), we encountered four midget RGCs with S-OFF responses and single cone receptive field centers (Figs 4B). A temporally-modulated S-cone isolating square-wave was used to assess S-cone inputs for every RGC encountered. The S-cone isolating stimulus modulates S-cone activity while holding L- and M-cones constant^43^. We experimentally validated the S-cone isolating stimulus at the beginning of each experiment by ensuring parasol RGCs did not respond to S-cone isolating modulations (Fig. 4A). As shown in Fig. 4B, each candidate S-OFF midget RGC responded robustly to the S-cone decrements. The tonic responses in Fig. 4B are characteristic of OFF midget RGCs and distinct from other cell types, such as the OFF parasol RGC in Fig. 4A.

**Figure 5:**
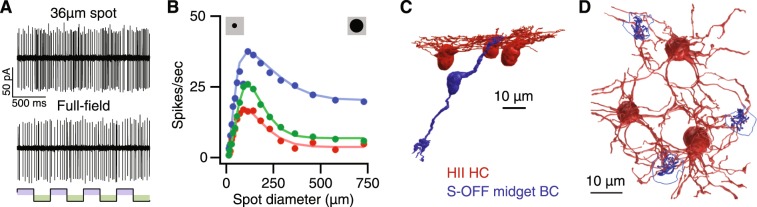
S-OFF midget RGCs encode spatial information with a center-surround receptive field. (**A**) Responses of an S-OFF midget RGC to a 36 µm spot vs a full-field stimulus (both S-cone isolating square-wave at 2 Hz). (**B**) Responses of an S-OFF midget RGC (blue) and two control L/M-cone midget RGCs (red and green) to achromatic spots of increasing diameter presented as temporal modulations (4 Hz square-wave). Smooth curves are fits to a Difference of Gaussians model. (**C**) The S-OFF midget receptive field obtained by the Difference of Gaussians fit in B. (**D**) The anatomical basis for the S-cone center-surround receptive field. The center receptive field represents the single S-cone input directly to the S-OFF midget BC (blue). HII horizontal cell feedback (red) forms the antagonistic surround receptive field. Scale bar is 10 µm.

The S-cone response kinetics were characterized using a time-varying “white noise” stimulus where the S-cone contrast of each frame was drawn pseudo-randomly from a Gaussian distribution (mean = 50%, SD = 30%; Fig. 4C). Convolving the stimulus with the elicited spike rate histogram in the Fourier domain returns a ‘filter’ that captures the linear components of the response. In other words, the linear filter represents the average S-cone modulation during the time preceding a spike and is proportional to the neuron’s impulse response function^44,45^. The average negative peak at 25 ms in Fig. 4C indicates that the neuron fires following decrements in S-cone contrast, the defining characteristic of an “S-OFF” neuron.

Our anatomical data confirm that HII horizontal cells carry both L/M- and S-cone signals (Fig. 2B)^29,46^. While an HII horizontal cell mediated L/M-cone surround has been reported in individual S-cones^47^, feedback from the strong S-cone surround has not been reported. Consistent with the presence of HII horizontal cell-mediated S-cone feedback, the S-OFF midget RGCs responded weakly to large, full-field S-cone isolating stimuli, preferring small spots centered over the receptive field (Fig. 5A). The weak responses to full-field stimuli may help explain why previous electrophysiology studies did not find evidence of S-OFF midget RGCs^11^.

The center-surround receptive field structure was investigated using an expanding spot stimulus temporally modulated in luminance (Fig. 5B). The spike rate increased as the spot size expanded to cover the entire center receptive field, then began decreasing as the spot further expanded to cover more of the antagonistic surround receptive field. The tuning profile of spatial opponency is comparable to L/M-cone OFF midget RGCs and clearly distinguished S-OFF midget RGCs from melanopsin RGCs, the other known S-OFF neuron in the primate retina^48^. Taken together, the S-OFF midget RGC anatomy and physiology indicates a center-surround receptive field, similar to L/M-OFF midget RGCs (Figs 4C, 5A–D).

The relationship between center-surround receptive fields, spatial information and edge detection is well-established^49–52^. Thus, the presence of center-surround structure in S-OFF midget RGCs indicates that, regardless of spectral tuning, the S-OFF midget RGC is carrying spatial information.

## Discussion

Here, we resolve a long-standing controversy and confirm the existence of S-OFF midget RGCs in the macaque retina. In addition, our work provides the most comprehensive reconstruction of the primate outer retinal S-cone circuitry to date, including the first complete reconstructions of horizontal cells in the primate retina.

We find that every cone in the central retina provides the sole input to an OFF midget bipolar cell. The pure S-cone midget RGC centers are not observed in the peripheral retina. There, OFF midget RGCs collect input from all nearby OFF midget bipolar cells, regardless of their cone contacts^2,53^. We propose that S-OFF midget RGCs should not be considered distinct from L-OFF and M-OFF midget RGCs. In the central retina, OFF midget bipolar cells draw indiscriminately from single cones regardless of type and, in the peripheral retina, where each midget RGC draws from multiple midget bipolar cells, the centers become mixed.

We find that S-OFF midget RGCs have the same center-surround receptive field structure as L vs. M midget RGCs. This receptive field structure has been characterized as optimal for the edge detection required for spatial vision^49^. It has been argued that edge detection must be achromatic and any degree of spectral opponency is detrimental^54,55^. However, not all edges are defined by changes in intensity alone, and equiluminant edges are common in natural scenes^56^. Thus, the spectral opponency in S-OFF midget RGCs could be used to signal the presence of an edge defined not only by a change in intensity, but also wavelength (reviewed by Patterson *et al*.^25^). S-cones contribute to the detection of white-yellow and gray-brown boundaries^57^, and are necessary when these boundaries are equiluminant. Both involve S-cone decrements, so S-OFF midget RGCs are well-suited for signaling brown or yellow objects against neutral backgrounds. In addition, seeing gray objects against the blue sky could be mediated by S-OFF RGCs.

A role in color perception is often the default hypothesis for cone-opponent neurons, particularly those with S-cone input. Indeed, S-OFF midget RGCs have been proposed to mediate yellow hue percepts, forming the OFF counterpart to the small bistratified RGC^58^, despite extreme asymmetries between the two types. However, there is no evidence that the color and spatial information from OFF-midget RGC are ever separated in our visual system. S-OFF midget RGCs may have evolved only to signal edges of objects against their background, particularly edges principally defined by spectral contrast between short and long wavelengths and they may have no role in generating color percepts.

Moreover, the S vs. L+M spectral tuning in both small bistratified and S-OFF midget RGCs does not match the cone inputs to blue-yellow hue perception^59–64^ and a subset of neurons with cone inputs matching the fundamental hues^65–68^ have been proposed to mediate hue perception instead (for review, see Neitz & Neitz^69^). The L vs. M midget RGCs with S-cone input are another controversial midget RGC subtype that warrants additional investigation. The significant technical challenges involved in studying rare S-cone inputs to parafoveal midget RGCs that may have limited these investigations in the past may now be overcome with the multi-disciplinary approach described here.

## Methods

All methods were performed in accordance with the relevant guidelines and regulations.

### Serial electron microscopy

#### Microscopy

The tissue was imaged using a Zeiss Sigma VP field emission scanning electron microscope equipped with a 3View system and sectioned in the horizontal plane. In 2018, the system was updated with an OnPoint™ detector that has been optimized for backscattered electrons (BSE) (Gatan, Inc.). All the EM images were obtained with the new detector. Optimized for biological samples, the OnPoint™ detector provides high BSE collection efficiency which translates to a favorable signal-to-noise ratio for visualizing small, low contrast features such as synaptic ribbons that have previously been a challenge for Serial block-face scanning. In each 70 µm section, an area approximately 200 µm on a side was imaged as a 5 X 5 montage at a resolution of 7.5 nm/pixel. The volume contained 1893 horizontal 90 µm sections from the ganglion cell layer through the cone pedicles. Image registration was performed using Nornir (http://nornir.github.io). The transmission EM image in Fig. 1A was taken of the inferior retina volume prior to sectioning.

#### Tissue Preparation

Retinal tissue was obtained from a terminally anesthetized male macaque (*Macaca nemestrina*) monkey through the Tissue Distribution Program at the Washington National Primate Center. All procedures were approved by the Institutional Animal Care and Use Committee at the University of Washington. A block of inferior parafoveal retinal tissue at ~1 mm eccentricity from the fovea center was processed as previously described^70^. Briefly, the eyecup was placed in 4% glutaraldehyde in 0.1M sodium cacodylate buffer pH 7.4 and while in this solution a 1 mm square of retina centered 2 mm temporal to the center of the fovea was cut out and then fixed overnight at 4 °C. The tissue was washed 5 × 5 minutes in 0.1M coacodylte buffer, then post fixed in osmium ferrocyanide for 1 hour on ice. The tissue was next washed 5 × 5 minutes in double distilled (dd)H2O at room temperature (RT) and incubated in a 1% thiocarbohydrazide solution for 20 minutes at RT. The tissue was washed 5 × 5 minutes in ddH2O and placed in 2% osmium tetroxide for 30 minutes at RT. The tissue was next washed 5 × 5 minutes in ddH2O and en block stained in 1% uranyl acetate, (aqueous), overnight in the refrigerator. The next day the tissue was washed 5 × 5 minutes in ddH2O, then en bloc stained in Walton’s lead aspartate for 30 minutes at 60 °C. The tissue as next washed 5 × 5 minutes in ddH2O and dehydrated in ice cold 30%, 50%, 70%, and 95% ETOH, then allowed to come to RT. This was followed by 2 changes of 100% ETOH and two changes of propylene oxide. The tissue was then infiltrated in a 1:1 mixture of propylene oxide:Durcupan resin, for 2 hours and then overnight infiltration in fresh Durcupan. The next day the tissue was given a fresh change of Durcupan for two hours and then placed in flat embedding molds and polymerized in a 60 °C oven for two days. The block was then trimmed to approximately 0.5 mm^2^. At this eccentricity (the edge of the foveal slope), the displacement of RGCs from cone pedicles was minimized while still remaining in a region where most midget RGCs receive single cone input.

#### Annotation

The serial EM volumes were annotated using the web-based, multiuser Viking software described previously^71^ (http://connectomes.utah.edu). Briefly, processes were traced through the sections by placing a circular disc at the structure’s center of mass and linking the disc to annotations on neighboring sections. Cone pedicles were outlined using a closed curve polygon defined by three or more control points. Synapses were annotated with lines connected by 2–3 control points and linked to a parent neuron. Synapse identification used previously described parameters^2,72^.

### Data analysis and visualization

Data analysis and 3D rendering were performed using an open-source Matlab program (https://github.com/neitzlab/sbfsem-tools)^73^. The cone pedicle analyses were based on XYZ coordinates of the closed curve control points, connected by Catmull-Rom splines. All other analysis was performed using the X, Y, Z coordinates and radius of the Disc annotations. For Fig. 1D, the closed curve coordinates were used to build a volume from which isosurfaces were extracted using the marching cubes algorithm and rendered as a triange mesh^74^. All other 3D models are triangle meshes built by rendering segments of connected annotations as rotated cylinders centered at each annotations’ XYZ coordinates and scaled by their radii.

Soma diameter was calculated from the single largest annotation in each neuron, assumed to be the soma. Primary dendrite diameter was calculated as the median diameter of annotations centered 0.5–1.5 µm from the soma.

The probability that the reported distribution of ribbon synapses in Fig. 3G was drawn by chance from a single normally-distributed group of L/M-cone OFF midget bipolar cell ribbon synapses was determined using a bootstrapping procedure. A normal distribution with the mean and standard deviation (SD) of both groups combined formed the null hypothesis. Seven integers were drawn from this normal distribution, then divided into two groups above and below the mean. The average SD of these two groups provided a metric for the degree of bimodality – two groups drawn from a normal distribution should have large SDs while two groups from two distinct distributions should have smaller SDs, as demonstrated by the clustering in Fig. 3G. The percentage of 10,000 boostrap ribbon synapse distributions with equal or higher average SDs than the original dataset determined the reported p-value. All other reported statistics used the Mann-Whitney-Wilcoxon ranked sum. The code and data used to generate the figures in this study will be made available on GitHub upon publication.

### Electrophysiology

#### Tissue preparation

Retinal tissue was obtained from terminally anesthetized macaque monkeys (*M*. *nemestrina*, *M*. *fasicularis*, *M*. *mulatta* of both sexes) through the Tissue Distribution Program at the Washington National Primate Center. All procedures were approved by the Institutional Animal Care and Use Committee at the University of Washington. Dissections were performed as previously described^75^. Briefly, enucleated eyes were hemisected and the vitreous humor was removed mechanically. When necessary, the eye cup was treated for ∼15 minutes with human plasmin (∼50 µg/mL, Sigma or Haematologic Technologies) to aid vitreous removal.

#### Recording

A piece of macaque macular retina with well-attached retinal pigment epithelium was placed on the stage of a microscope ganglion cell side up. The tissue was superfused with warmed (32–35 °C) Ames’ medium (Sigma) at ∼6–8 mL min^−1^. In some cases, additional D-glucose (14 mmol) was added to the Ames’ medium^76^. Ganglion cell spikes were measured with extracellular or loose-patch recordings using an Ames-filled borosilicate pipette. The data was sampled at 10 kHz (Multiclamp 700B, Molecular Devices), Bessel filtered at 3 kHz and digitized using an ITC-18 analog-digital board (HEKA Instruments).

#### Visual Stimuli

Stimulus presentation and data acquisition used the open source programs Stage (www.stage-vss.github.io) and Symphony (www.symphony-das.github.io), respectively. The Symphony stimulus protocols used in this study can be found at https://github.com/sarastokes/sara-package. Visual stimuli were projected onto the cone outer segments through a 10x objective (Olympus) using a Lightcrafter DLP 4500 (Texas Instruments) with a 60 Hz frame rate. To optimize S-cone isolation, the built-in LEDs were replaced with custom LEDs at 405, 535 and 630 nm. The 405 nm primary produces relative quantal catches in S-, M-, and L-cones of 1.0, 0.20 and 0.21 respectively and thus, when used alone is about 80% S-cone isolating. This offers advantages in generating S-cone isolating stimuli over the standard 456 nm blue primary in the Lightcrafter DLP 4500 (Texas Instruments) which drives L- and M-cones more strongly with relative quantal catches of S-, M-, and L-cones of 1.0, 0.57 and 0.35 respectively.

Each LED was calibrated by measuring the spectral distribution with a spectroradiometer (Konica Minolta CS-2000) and the power with an optometer (UDT-300). A transformation matrix, *A*, relating the LED weights to the cone quantal catches was obtained by taking the outer product of the LED spectra (*R*(λ), *G*(λ) and *B*(λ), as only three of the four LEDs were used at a time) and the L-, M- and S-cone spectral sensitivities (*L*(λ), *M*(λ), *S*(λ) respectively):

$${\rm{A}}=[\begin{array}{ccc}{L}_{R} & {M}_{R} & {S}_{R}\\ {L}_{G} & {M}_{G} & {S}_{G}\\ {L}_{B} & {M}_{B} & {S}_{B}\end{array}]=[\begin{array}{ccc}L({\rm{\lambda }}) & M({\rm{\lambda }}) & S({\rm{\lambda }})\end{array}]\ast [\begin{array}{c}R({\rm{\lambda }})\\ G({\rm{\lambda }})\\ B({\rm{\lambda }})\end{array}]$$

The transformation matrix was used to solve for the appropriate LED weights for any given level of L-, M- and S-cone activations^43^.

The mean light levels were calculated using a collecting area of 0.37 µm^2^ and 1 µm^2^ ^77^. All stimuli used photopic light levels (∼3 × 10^3^ to 3 × 10^5^ R*). To maintain a constant state of light adaptation, the mean light level was displayed continuously between stimulus presentations. Contrast is expressed as Weber contrast.

### Cell Identification and Selection

RGCs were initially identified by soma appearance, as visualized with a 60x objective (Olympus) under infrared illumination. RGC type was further determined by responses to spots, cone-isolating stimuli and mapping the receptive field with horizontal and vertical bars. Midget RGCs make up over 90% of all RGCs in the central retina^78^ and were confirmed by small soma, sustained responses and small center-surround receptive fields^79,80^. OFF RGC somas were generally vitread to ON RGC somas. In addition to these criteria, S-OFF midget RGCs were identified using small S-cone isolating stimuli positioned over the receptive field center.

### Recording protocol

Tissue sensitivity was assessed at the beginning of each experiment by ensuring ON parasol RGCs responded to a full-field (~1 mm), 5% contrast, 4 Hz temporally-modulated spot^81^. Parasol RGCs lack significant S-cone input and were also used to validate the S-cone isolating stimuli (Fig. 4A)^8^.

For each subsequent RGC encountered, the polarity (ON, OFF or ON-OFF) and cell type were first determined by spots presenting high contrast luminance increments and decrements from a photopic mean light level. Cell type was confirmed by receptive field dimensions. The receptive field center was determined by vertical and horizontal bars, presented as 2–4 Hz squarewave temporal modulations. The center and surround receptive field radii were measured from Difference of Gaussian fits to expanding spots and annuli. In cases where the receptive field dimensions were unclear (either due to noise or a rare cell with an atypical receptive field), the measurements were confirmed by estimating the spatiotemporal receptive field using coarse (25–50 µm square pixels) binary spatial noise.

S-cone contributions were measured in each RGC with different sizes of S-cone isolating spots, presented as 1–2 hz squarewave temporal modulations. For each RGC with a significant S-cone response, the temporal and spatial characteristics were next measured with S-cone isolating full-field temporally modulated Gaussian noise and drifting gratings, respectively.

Expanding spot: High contrast luminance spot stimuli were positioned at the receptive field center and presented as a 4 Hz temporal modulation.

Gaussian noise: The temporal ‘white noise’ stimulus was generated by psuedo-random draws from a Gaussian distribution centered at the mean light level (mean, 50%, SD = 30% contrast). The noise stimulus was presented in 10 or 20 second epochs, with 1 second interval between epochs.

### Analysis

To extract the spike responses, recorded currents were high-pass filtered, then sorted using k-means clustering. In some cases, the returned spikes were further sorted by setting a manual amplitude threshold. Analysis of the moving edge and expanding spot stimuli were based on the F1 amplitude, the response at the modulation frequency (temporal for expanding spots, spatial for moving edges). The F1 amplitudes were calculated from the cycle-averaged spike rate, binned at 60 Hz. Unless otherwise specified, data are expressed as mean ± SEM. Significance was determined using the Mann-Whitney-Wilcoxon rank sum test. All analyses were performed in Matlab 2017b (Mathworks) and the final figures prepared in Igor Pro 7 (Wavemetrics, Oswego WA).

#### Difference of gaussians model

A Difference of Gaussians (DoG) model was fit to the F1 amplitudes^82,83^. The DoG model characterizes the center and surround receptive fields as two antagonistic, two-dimensional Gaussians with separate strengths and sizes. The center and surround receptive fields are assumed to be radially symmetric and centered at the same location. The DoG model predicts the response, *R*, for spot diameter, *f*, as:

$${\rm{R}}({\rm{f}})={{\rm{R}}}_{0}+({{\rm{K}}}_{{\rm{c}}}{{\rm{\pi }}{\rm{\sigma }}}_{{\rm{c}}}^{2}{{\rm{e}}}^{-{({{\rm{\pi }}{\rm{\sigma }}}_{{\rm{c}}}{\rm{f}})}^{2}})-({{\rm{K}}}_{{\rm{s}}}{{\rm{\pi }}{\rm{\sigma }}}_{{\rm{s}}}^{2}{{\rm{e}}}^{-{({{\rm{\pi }}{\rm{\sigma }}}_{{\rm{s}}}{\rm{f}})}^{2}})$$

where *R*_0_ *is* the baseline response, *σ*_*c*_ and *σ*_*s*_ are the center and surround receptive field sizes, respectively, and *k*_*c*_ and *k*_*s*_ are the center and surround strengths, respectively.

#### White noise analysis

Analysis was performed on the spike rate, binned at 360 Hz. The first second of the response was omitted to control for adaptation. The linear filter, *F*, is obtained by cross-correlating the stimulus, *s*(*t*), with the spike rate, *r*(*t*), and dividing out the stimulus’ power spectrum:

$$\tilde{{\rm{F}}}({\rm{\omega }})=\frac{\tilde{{\rm{s}}}\ast ({\rm{\omega }})\tilde{{\rm{r}}}({\rm{\omega }})}{\tilde{{\rm{s}}}\ast ({\rm{\omega }}){\rm{s}}({\rm{\omega }})}$$

where $\tilde{{\rm{s}}}({\rm{\omega }})$ is the Fourier transform of s(t), $\tilde{{\rm{r}}}({\rm{\omega }})$ is the Fourier transform of s(t) and * denotes the complex conjugate. In practice, the stimulus power spectrum was nearly flat and the denominator was omitted^84^. The inverse transform of $\tilde{{\rm{F}}}({\rm{\omega }})$ returned the time-domain linear filter, *F*.

## Data Availability

Access to the macaque EM volume dataset is available on request. Visualizing both the dataset and the annotations requires the Viking Viewer developed in Bryan Jones’ lab at (http://connectomes.utah.edu). The 3D reconstructions from Viking Viewer annotations are visualized with SBFSEM-tools, an open-source Matlab toolbox developed in the Neitz lab (https://github.com/neitzlab/sbfsem-tools). Access to the dataset through the Viking Viewer and SBFSEM-tools does not require that the user install the data locally. The data and code used to generate each figure will be made available on publication at https://github.com/neitzlab/SConeEdgeDetection.
